# Employer and Co-worker Perspectives on Hiring and Working with People with Mental Health Conditions

**DOI:** 10.1007/s10597-021-00934-2

**Published:** 2022-01-31

**Authors:** Shazana Shahwan, Zhang Yunjue, Pratika Satghare, Janhavi Ajit Vaingankar, Yogeswary Maniam, Goh Chong Min Janrius, Teh Wen Lin, Kumarasan Roystonn, Mythily Subramaniam

**Affiliations:** 1 Research Division, Institute of Mental Health, Buangkok Green Medical Park, 10 Buangkok View, Singapore, 539747 Singapore; 2 Department of Psychosis, Institute of Mental Health, Singapore, Singapore

**Keywords:** Work, Stigma, Mental illness, Employment

## Abstract

The purpose of this study was to understand perspectives towards hiring and working with people with mental health conditions (PMHC). Semi-structured interviews with 25 employers and 20 co-workers were carried out. Thematic analysis was used to analyse the data. The barriers to hiring and working with PMHC identified through the interviews were concerns about safety, incompetence, PMHC not being able to get along with others, requiring more training and supervision as well as medical costs and reputational risks to the hiring organisation. Employers and co-workers suggested that improving mental health literacy of staff, pairing the PMHC with trained work buddies, having access to mental professionals when needed, and providing incentives for hiring PMHC such as tax rebates are likely to improve attitudes towards hiring and working with PMHC. Their suggestions for the additional supports required should be considered when developing initiatives to promote inclusivity of PMHC in workplaces.

People with mental health conditions (PMHC) are typically “the last hired and the first fired” (Thompson et al., 2002). Individuals with severe and common mental health conditions are 7 and 3 times more likely to be unemployed compared to individuals with no such disorder, respectively (Hoedeman, 2012). An epidemiological study that investigated the influence of six common chronic conditions (inflammatory conditions, cardiovascular diseases, common mental disorders, respiratory illness, diabetes and psychotic disorders) on entering paid employment reported that the impact of a chronic disease on maintaining unemployment was the largest for mental disorders (Yildiz et al., 2021). Even after being successfully hired, Nelson and Kim found that individuals with mental illness have an increased risk of employment termination in general as well as both involuntary and voluntary job loss (Nelson & Kim, 2011). The primary barrier that stymies individuals from gaining entry and retaining their place in the workforce is stigma (Khalema & Shankar, 2014; Krupa et al., 2009). Even in the absence of aberrant behaviour or when the illness is in complete remission, PMHC face social discrimination and rejection due to the diagnostic labels attached to them (Thornicroft, 2006).

Various studies show that employers are largely uninformed about the relationship between mental health and work ability and tend to subscribe to stereotypes when making hiring decisions (Shankar et al., 2014). Krupa et al., in their effort to understand the forces that perpetuate employment-related mental illness stigma, identified 5 commonly-held assumptions within workplaces (Krupa et al., 2009). These included the (i) assumption of incompetence (ii) assumption of dangerousness and unpredictability, (iii) belief that mental disorder is not a legitimate illness, (iv) belief that working is unhealthy for people with mental illness, and (v) assumption that employing these individuals represents an act of charity inconsistent with workplace needs. These assumptions are sustained by broader social and structural systems such as negative media portrayals, the mental health system focusing on pathology and deficits while paying limited attention to employment needs of PMHC, and lack of policies guiding how employers might meet their obligations to PMHC (Krupa et al., 2009).

PMHC desire to work, and in turn, work itself confers numerous benefits to their recovery including attainment of financial gains, social connectedness and belonging, cognitive stimulation, self-identity and worth, as well as finding purpose and meaning in life (Blank et al., 2015; Subramaniam, Zhang, et al., 2020). Evidence supports that individuals with mental illness can work effectively and safely in a competitive environment with adequate support. According to the review by Bond, Drake, & Becker (Bond et al., 2008), the single best predictor of competitive employment for patients with schizophrenia is supported employment with the Individual Support and Placement (IPS) model being regarded as the gold standard. IPS is a systematic approach to helping people with severe mental illness attain competitive employment. It is based on eight principles: eligibility based on client choice, focus on competitive employment, integration of mental health and employment services, attention to client preferences, work incentives planning, rapid job search, systematic job development, and individualized job supports (Bond et al., 2012).

Focusing on preparing PMHC for job search, helping them find jobs, and supporting them on the job (i.e. supply-side strategies) have minimal impact on the rate of competitive employment among PMHC (Delman et al., 2017). Increasing attention is now directed at ‘demand-side’ strategies that focus on understanding the demands arising from the work environment and the organisation’s responses to PMHC, particularly the attitudes and behaviours of supervisors and co-workers (Delman et al., 2017). Some settings are naturally supportive to PMHC (e.g., when there is a good job-person fit, good relationship with supervisors and co-workers, flexible schedules etc.). When natural supports are not in place, mental health advocacy groups have argued that organisations should ensure that work accommodations are made for requests that are reasonable and feasible. Examples of such requests include those that do not slow down the productivity, do not cause undue hardship, or generate excessive costs (Corbière et al., 2014). A recent systematic review of work accommodations for people with mental disorders showed that these helped PMHC meet employment expectations with minimal cost (Zafar et al., 2019). However, an important issue related to attaining work accommodation is the disclosure of their condition in order to initiate discussions with supervisors. This creates a dilemma for PMHC given the potential negative consequences that follow disclosure.

In Singapore, there were no labour laws that prevented the discrimination of those with mental illness up till December 2019 when the Tripartite Alliance for Fair and Progressive Employment Practices (TAFEP) updated its guidelines stating that it is discriminatory to ask job applicants to declare their mental health condition unless there is a job-related requirement (Zhou, 2020). This recent local advancement trails behind that in countries such as the United States, whose anti-discrimination law date two decades prior (i.e., American Disabilities Act of 1990).

Not surprisingly, given the assumptions about PMHC described earlier, PMHC are highly overrepresented among the unemployed. The Singapore Mental Health Study 2016 revealed that the unemployment rate among those with a 12-month mental disorder was 11.5%, in comparison to 4.8% among those without a mental disorder (Subramaniam et al., 2021). Furthermore, an attitudes survey by the National Council of Social Service (NCSS) reported that only a little more than half of the respondents indicated that they were willing to live with a PMHC (Ng, 2018). Hence, to provide jobs for able and qualified PMHC, it is important to understand the concerns of co-workers and employers and what they require to address these concerns. The purpose of this study was thus to examine co-workers’ and employers’ perspectives towards people with mental illness, including their concerns about hiring PMHC and the support needed to hire them.

## Methods

### Study Design

This research was part of a larger mixed-methods study that examined the employment needs of young PMHC from a multi-stakeholder perspective. The study comprised semi-structured interviews with PMHC, their caregivers who provided informal support, employment specialists, and other professionals who provided formal support to help PMHC gain employment. The data for these stakeholder groups have been published earlier (Subramaniam, Zhang, et al., 2020; Teh et al., 2020; Vaingankar et al., 2020). Interviews were also conducted with employers and co-workers of young people who may or may not have had the experience of working with someone with a mental health condition, which is the focus of this study. In all, 130 interviews were completed between 2017 and 2018 and analysed. Of these, 45 interviews were conducted with co-workers and employers which were analysed and discussed in this study.

### Sampling and Participants

The study employed a purposive and maximum variation sampling (Patton, 2002) to select study participants who varied by age, gender, ethnicity, industry, and length of employment in the company. Employers were recruited through referrals by study team members and their colleagues as well as local databases with employer details. Individuals who were involved in hiring discussions and/or decisions in their respective departments or organisations were regarded as employers in this study. These individuals tended to hold management positions or were human resource personnel. Employers had to meet the following inclusion criteria: age 21 and above, speak English, work in any organisation and be in charge of employee recruitment. Co-workers were employees representing a range of jobs in various industries and were not considered ‘employers’ as defined above. They were recruited through referrals by study team members and their colleagues and snowballing. Co-workers had to meet the following criteria: age 21 and above, speak English, working in any organisation, and with or without prior experience of working with someone diagnosed with a mental health condition. Employers and co-workers in mental health or social services were not included in this analysis in order to reflect the views of individuals working in the broader open labour market. The views of professionals in mental health and social services providing employment-related support to PMHCs were analysed separately and published (Vaingankar et al., 2020).

In all 25 employers and 20 co-workers were recruited. Age, gender, ethnicity, and the number of years worked are presented in Table 1a and Table 1b for employers and co-workers, respectively. In addition, descriptions of each participant, including their occupation and industry served, are indicated in Table 2a and b for employers and co-workers, respectively.

**Table 1:** Demographic profile of employers and co-workers

a Demographic profile of employers			
Sociodemographic variables		N	Percentage
Gender	Female	18	72
	Male	7	28
Age	24–35	14	56
	36–45	5	20
	≥ 45	6	24
Ethnicity	Chinese	22	88
	Indian	1	4
	Malay	1	4
	Eurasian	1	4
Education	Diploma and Below	9	36
	University and Above	16	64
Marital status	Married	14	56
	Not Married	11	44
Working years	< 3	7	28
	< 10	14	56
	≥ 10	4	16
Organization size^#^	≤ 30	10	40
	≤ 200	8	32
	≥ 200	6	24

b Demographic profile of co-workers			
Socio-demographics variables		N	Percentage
Gender	Female	9	45
	Male	11	55
Age	24–35	17	85
	36–45	2	10
	≥ 45	1	5
Ethnicity	Chinese	12	60
	Indian	6	30
	Malay	2	10
Education	Diploma and Below	8	40
	University and Above	12	60
Marital status	Married	11	55
	Not Married	9	45
Working years	< 3	13	65
	< 10	5	25
	≥ 10	2	10

*Working years ranges from 2 months to 20 years in Table a

*Organisation size ranges from 6 to 1300 employees

^#^Percentages do not add up to 100% due to 1 missing data

*Working years ranges from 5 months to 20 years in Table b

**Table 2:** Age and occupational background of employers and co-workers

Participant no	Age	Industry	Occupation	Working years
a Age and occupational background of employers				
EP 01	24	Education	HR Executive	2
EP 02	26	Medical	Clinic Manager	2
EP 03	42	Market Research	Director	6
EP 04	48	Manufacturing	HR Executive	3
EP 05	35	Entertainment/Tourism	HR Manager	3
EP 06	55	Information Technology	HR Executive	2.5
EP 07	27	Manufacturing	Group HR Manager	3
EP 08	52	Education	Tuition Centre Manager	8
EP 09	35	Healthcare	Clinic Manager	9
EP 10	41	Insurance	HR Manager	10
EP 11	30	Shipping	HR Executive	2
EP 12	42	Research Consultancy	Director	8
EP 13	29	Manufacturing	Chemist	3
EP 14	29	Service	HR Executive	2.5
EP 15	35	Public Service	HR	12
EP 16	25	Recruitment Agency	HR Manager	3.5
EP 17	52	Service	Club manager	24
EP 18	47	Information Technology	Manager	< 1
EP 19	30	Entertainment/Tourism	Director	3
EP 20	30	Maritime	HR Business partner	3
EP 21	33	Recruitment	Sales Coordinator	< 1
EP 22	44	Manufacturing	HR Manager	5
EP 23	30	Entertainment/Tourism	Director	6
EP 24	38	Business Consultancy	Accounting, HR	4
EP 25	58	Manufacturing	Executive administrator	20
b Age and occupational background of co-workers				
CW 01	27	Retail	Graphic Designer	≤ 3
CW 02	28	Information Technology	Consultant	5.5
CW 03	29	Logistics	Manager	≤ 3
CW 04	27	E-Commerce	Executive	≤ 3
CW 05	40	Banking	Bank Officer	20
CW 06	40	Manufacturing	Despatch	6
CW 07	26	Insurance	Financial Consultant	≤ 3
CW 08	30	Business Consultancy	Account Manager	≤ 3
CW 09	24	Retail	Marketing Officer	≤ 3
CW 10	33	Construction	Project Manager	≤ 3
CW 11	48	Shipping	Manager	10
CW 12	29	Marketing	Senior Marketing Executive	≤ 3
CW 13	29	Ministry	Associate	≤ 3
CW 14	33	Construction	Project Engineer	6
CW 15	32	Ministry	Allied Educator	6
CW 16	30	Marketing	HR executive	≤ 3
CW 17	25	Electronics	Executive Specialist	≤ 3
CW 18	29	Information Technology	System analyst	≤ 3
CW 19	35	Military	Senior consultant	8
CW 20	33	Shipping	Logistics officer	≤ 3

### Interview Procedures

Face-to-face in-depth interviews were conducted with the participants at a mutually agreed upon venue and time. The interviews were conducted by trained qualitative interviewers (MS, JV, CZ, SS and PS). Participants also completed brief demographic data forms. Each interview lasted between 1 and 1.5 h. Interviews were conducted using an interview guide (see Table 3a and b for topics covered). Neutral probes were used to encourage participants to elaborate and provide examples from their own experiences. Interviews were conducted in English and transcribed verbatim. All participants were well-versed in English. Recruitment of participants and the interviews were carried out till data saturation was reached. Participants received an inconvenience fee of SGD 60 after completing the interview.

**Table 3:** Interview guide for employers and co-workers

Section	Questions	
a Interview guide for employers		
Background of co-workers work and organisation	•Can you tell me a bit about yourself? Are you directly involved in hiring personnel in your organisation? •Can you tell me about your organisation?	
Perspectives of working with a PMHC	•What are your thoughts regarding employing young people with mental health conditions (PMHC)? Can you tell me why you think this way? •Have you ever employed anyone with mental health conditions or do you have any experience of dealing with PMHC at the workplace?	
	If **Yes,** •Can you tell us about any positive and/or negative experiences associated with this person?	If **No,** •Would you consider employing PMHC? Can you tell me why you feel this way? •What would you do if you find out that one of your workers had a mental health condition? Can you tell me why you would feel this way?
	•What do you think are some of the barriers and challenges to employing a PMHC in your organisation? Can you tell me why there are these barriers and challenges? How might they be overcome? •How do you think the co-workers would behave/ feel if they knew that a PMHC is working with them? Why do you think they would behave/ feel this way? •Has a co-worker ever highlighted any problems of working with a PMHC to you? Can you tell me more about it? How did you deal with it? Why did you choose to deal with it this way?	
Workplace mental health initiatives and support needed for PMHC	• Does your organisation have any mental wellness initiatives? • At an organisational level what support do you think you or your employees would need to employ PMHC? • Are you aware of any support services that can help PMHC with their employment needs?	
b Interview guide for co-workers		
Background of co-workers work and organisation	Can you tell me a bit about yourself and your work? Can you tell me about your organisation?	
Perspectives of working with a PMHC	What are your thoughts regarding working with PMHC? Why do you feel this way? Have you ever worked with anyone with mental health conditions or do you have any experience of dealing with PMHC at the workplace?	
	If **Yes,** Can you tell us about any positive and/or negative experiences associated with this person?	If **No,** Would you consider working with PMHC? Can you tell me why you feel this way? What would you do if you find out that one of your colleagues had a mental health condition? Why?
	Has anyone ever highlighted any problems of working with a PMHC to you? Can you tell me more about it?	
Workplace mental health initiatives and support needed for PMHC	Does your organisation have any mental wellness initiatives? Is there any section or discussion related to mental health conditions in them? What do you think is needed to make people feel more comfortable about working with PMHC?	

### Coding and Data Analysis

Audio recordings of the interviews were transcribed and imported into NVivo 11 software for purposes of coding and analysis. Thematic analysis was used to analyse the data. The authors involved in the coding (MS, SS, CZ, and PS) read and re-read the initial five employer transcripts to identify potential themes. This process of “repeated reading” (Braun & Clarke, 2006), resulted in data immersion and ensured the researcher’s closeness with the data. The themes were then provided to the first author. The second level of analysis involved these authors reviewing the basic themes and finalizing them based on the frequency and similarity of responses. These themes were then used to develop a codebook that had been mutually agreed on by all the coders. The codes were specified with the following: label, definition, inclusions and exclusions, and typical and atypical exemplars from the raw data. Coding of the same transcript by the four coders commenced after the first draft of the codebook was finalized for the purpose of achieving optimum inter-rater reliability. This process was reviewed and repeated with a different transcript until Kappa scores above 0.70 was reached.

Once optimum inter-rater reliability was achieved, the transcripts were randomly assigned to the specified coders. Further coding also took place at this stage to ensure no themes had been missed in the earlier stages. If new themes emerged, they were highlighted and included by all the coders. After coding all the transcripts, the coders met again to discuss and decide how to organise the themes. The final themes were decided by eliminating redundancies, discarding minor or irrelevant themes, merging closely related major themes, and applying a structure of global, organising, basic and sub- themes (Gee & Skovdal, 2017). The above processes were repeated for the co-worker transcripts. The employer and co-worker interviews were analysed together to provide a more comprehensive account of perspectives regarding potential or actual encounters with PMHC in workplaces. Themes were examined for convergences, divergences, and regularities in the data, and where there were differences in responses from the two stakeholder groups; these have also been highlighted in the findings reported below.

## Results

Three organising themes pertaining to employer and co-workers’ perspectives towards hiring and working with PMHC were identified. They were (1) Facilitators and barriers to hiring and working with PMHC, (2) Pros and cons of workplace disclosure, and (3) Workplace initiatives to promote mental health and inclusivity of PMHC. The basic and sub-themes of each of these organising themes and the number of employers and co-workers who provided responses supporting each sub-theme identification are presented in Table 4. These themes are further described below.

**Table 4:** Frequency of participants supporting sub- themes of employer and co-worker perspectives towards hiring and working with PMHC

Basic themes	*Sub-themes*	Number of employers	Number of co-workers
(1) Facilitators and barriers to hiring and working with PMHC			
a. Facilitators	*Ability to work prioritised over mental health condition* *Helping PMHC get back on their feet*	17 10	10 14
b. Barriers	*PMHC-oriented concerns* *Co-worker- and employer- oriented concerns* *Jobs deemed suitable for PMHC* *Expectations that PMHC should work at lower pay scale*	16 11 14 3	15 9 10 0
(2) Pros and cons of disclosure			
	*a. Pros*	14	3
	*b. Cons*	9	5
(3) Workplace initiatives to promote mental health and inclusivity of PMHC			
	*a. Existing initiatives*	11	13
	*b. Suggested workplace support for PMHC*	18	0

### Facilitators to Hiring and Working with PMHC

Co-workers and employers believed in the value of work for PMHC at an attitudinal level. Although the number of barriers described later far outweighed the facilitators, the positive attitudes represents the openness of co-workers and employers to consider a PMHC in the workforce.

### Ability to Work Prioritised Over Mental Health Condition

Several co-workers expressed that they trusted and respected the decisions of those in hiring positions in their organisations and were supportive if PMHC were selected.

I mean, he’s obviously hired for a reason; if the management deems that he’s ok to work here, I think it’s perfectly fine for me (CW13/F/29)

From the perspective of the employer, a PMHC whose condition was well managed through treatment and outperformed other applicants should be given a chance to prove themselves.

……she’s on medication, and then you know maybe you know if she’s (an) exceptionally bright person you know from the CV, and then you know we feel that she can contribute with her good ideas and creativity, then maybe we will just give her an opportunity to prove herself. (EP17/F/52)

Both co-workers and employers reasoned that employment becomes essential for practical reasons as PMHC had roles and responsibilities just like them.

I mean they also deserve a chance to make a living, and they are facing their own internal struggles and what not and if there is something that is controllable, and doesn’t cause harm to anybody then like I don’t think there is any reason why to like … like be bias against them. (CW08/F/30)

Other employers highlighted anti-discrimination policies that their company had and thus would not ask whether the applicant had a mental illness.

So, we don’t actually ask about all these because to us that is irrelevant and it’s not necessary to come and do the job… The tripartite employment services and all that, so we have actually signed-on to pledge with them, because already we are hiring in a way in accordance with what they believe in, and that includes not disclosing a lot of things for hiring. (EP05/F/35)

### Helping PMHC Get Back on Their Feet

Several co-workers and employers recognised that PMHC may not have the skill set required at the point of hire but by giving them an opportunity, they would be able to contribute in the workplace.

I mean same for the person with a mental health condition. I think with more confidence and a bit more practice right, he or she might be able to handle it herself after a while ready. (CW12/M/29)

Others demonstrated their willingness to support a PMHC where needed.

I’ll try to get to know the person and see how I can help the person at work, yeah. And then if I see that in any way the person needs help, I’ll definitely help. (CW17/F/25)

### Barriers to Hiring and Working with PMHC

The concerns raised by co-workers and employers regarding hiring and working with PMHC could be classified into two broad categories: PMHC oriented concerns pertained to unfavourable perceptions and experiences that co-workers and employers held towards PMHC in the work setting; whereas Co-worker and Employer oriented concerns related to the staff’s insights on their limitations in supporting a PMHC in the workplace. Two additional sub-themes that may pose as barriers to hiring PMHC were ‘jobs deemed suitable for PMHC’ and ‘expectations that PMHC should work at a lower pay scale’.

### PMHC-Oriented Concerns

Employers and co-workers were concerned about emotional outbursts that could pose risks of harm to other co-workers and clients.

maybe violence that’s really taking it to the extreme then they might also be concerned, worried about safety for themselves … yeah, because think we associate that mental health condition has some form of um… instability in the person yeah…so I guess he might have some concerns with safety as well. (CW13/F/29)

They were also worried about the ramifications if PMHC were to endanger themselves.

Behaviours that can be recurring and maybe damaging to themselves as well as the people around them. I mean repetitive, I don’t mean like one. So if it’s too repetitive, to the point that we have to constantly mitigate? Then unfortunately, they will be let go at the end. (EP05/F/35)

Employers and co-workers envisioned or had experienced instances when PMHC demonstrated poor work ethics such as expressing a “can’t do” attitude and were unreliable.

So I was like ok let’s make it happen, then suddenly this guy disappears on me. Then I feel a bit, very annoyed actually. (CW04/M/27)

They were worried that PMHC incompetence would result in other staff shouldering the PMHC’s responsibilities on top of their own workload.

I can see the…a bit of a pattern, like he will break down every few months then come back up and then something else will happen every few months that affect us and the operations. (EP05/F/35)

Moreover, employers and co-workers were concerned that PMHC may be rigid in their ways.

It’s very difficult to get them to do teamwork. They really have barriers, very difficult, they have a lot of own mentality ways. (EP04/F/48)

They were also worried that PMHC’s emotionality and unreasonable behaviour may give rise to unpleasant working dynamics.

She will act sometime very abnormal just throw out a temper and then just walk out of the shop, and then ask you to take over the shop…and then the workers, the staff actually not very happy with her…… (EP04/F/48).

### Co-worker & Employer Oriented Concerns

Some co-workers expressed that they may lack the skills and ability to support PMHC at work. These concerns were related to having the patience to guide the PMHC as they perceived PMHC to lack cognitive ability, require more instructions, training and supervision than the average worker.

So I don’t know what my tolerance level will be also, it’ll be like handling a small child la I think, about 2–3 years old. (CW20/M/33)

They also expressed that they would have to be mindful of how they spoke, and not to over-stress them with work, indicating that they perceived PMHC as requiring special treatment and a need to be cautious around them.

Now the situation is that they treat her better not in the sense of care and concern, but rather just to don’t provoke her, stay out of her way, maybe not do anything. (EP09/F/35)

Employers and co-workers also expressed apprehension that hiring a PMHC could be expensive to the company in terms of medical bills.

Ok…the risks like…is there a tendency for this person to…for relapse of the medical…of the condition? OK, then, also the costs involved if he’s sick, you know…the company got to bear the cost, the medical cost. (EP06/F/55).

Additionally, they feared the company’s image being tarnished if the PMHC were to represent the company poorly.

……basic job I think can, because if you do accounts for customers, there’s a risk the person do wrongly or what, because ya…it may affect the company’s reputation. (EP06/F/55)

### Jobs deemed suitable for PMHC

In line with the concerns raised about hiring PMHC, co-workers and employers suggested specific jobs that they felt were suitable for PMHC. These jobs tended to be manual, repetitive, entry level jobs,

I think shredding paper. I don’t think there is a lot of paper to shred…er (long pause) …maybe sanitation-related jobs, cleaning, helping to clear things properly. (CW07/M/25)

back-end administrative duties,

Probably if it’s some work with regards to the backroom, then it shouldn’t be a problem. Backroom duties as in admin, filing, then that work will be able to suits them better? (CW05/F/40)

or those in social service sectors that specifically hire PMHC or in the food and beverage industries.

Yes, it has to start somewhere, so you know; it can be like in government organisations or like you know Fast-food chains and all that. It has to start somewhere. (CW12/M/29)

Jobs that were identified as unsuitable were those at the frontline especially those which involved serving vulnerable populations, were fast-paced and stressful, cognitively demanding and where mistakes would result in dire consequences for the company.

……I would think probably not customer facing because you would not know what these customers may want. Some customers are pretty nasty. (EP23/M/30)

### Expectations That PMHC Should Work at a Lower Pay Scale

Three employers highlighted that if a PMHC were willing to accept a lower salary, the organisation may view them more favourably.

To be blunt la okay if both are degree holder, one have mental health condition and they don’t mind lowering down their salary like intake right salary amount I think the company will be more than happy to want to try to accept that person. (EP13/F/29)

These employers viewed such an approach as benefiting both the organisation as well as the PMHC.

Now, mind you I am not exploiting them, if I propose to the management for lowest salary range of staff, but rather this is the bridging opportunities for them. (EP09/F/35)

## Pros and Cons of Disclosure

Although there were no specific questions in the interview guide regarding the pros and cons of disclosure, themes surrounding disclosure emerged spontaneously.

### Pros

Employers and co-workers preferred to be informed of mental health conditions of employees for transparency, to be able to empathise, and to prepare themselves if an incident were to occur.

I do think is also my responsibility to highlight to the hiring manager who is responsible in the day-to-day affairs of the employee, so that the person is aware, the person can better manage if the employee is being hired. (EP10/F/41).

### Cons

While disclosure of an individual’s mental health condition was preferred, employers admitted that this may cost PMHC any chances of being hired. Forthrightly, employers and co-workers shared that even if they were aware of the condition, there were no guidelines in the organisation to support PMHC that they were aware of. Rather this knowledge may only serve to alienate the PMHC from others.

…it’s still quite a taboo topic is because I guess most of us don’t have the solutions for it. (CW13/F/29)

Due to the discrimination and rejection that PMHC may face, a small group of employers believed that the decision to disclose rests entirely on employees. To them, mental health is a private and personal matter.

During the interview we don’t ask, cos I think this is also like part of the fair employment practice act. (EP14/F/29)

## Workplace Initiatives to Promote Mental Health and Inclusivity of PMHC

### Existing Mental Health Promotion Activities in Workplaces

Many employers and co-workers talked about three main kinds of health and wellness promotion activities in their workplaces. These were not specific to PMHC and were offered to all staff. These took the form of:

(i) short workshops

Actually my boss signed up for workplace health, so the whole year we had wellness program, health and wellness program, talks on like how to de-stress you know, exercises to de-stress. Talks on how to manage stress and things like that. So we also have exercises like Yoga all these things, part of the WHP program. (EP06/F/55).

(ii) one-to-one informal sessions with their supervisors

Mental wellness, we do it on a more, what is it called, it’s a more individual basis like the managers and directors will speak to their members individually to check their status and see how they are doing, whether they are coping …it’s like a one-to-one meeting like the bank manager or director will just meet up with me for a meal, one-to-one, the director much lesser time because he has more things to manage ma, to speak to so the manager will assume a quarter of the load so she’ll meet up with us one-on-one. (CW07/M/26).

and (iii) access to hotlines or counsellors which were part of the employee assistance programmes that staff could access if needed.

Every employee actually has an access to a number that they can call to approach a phone … a counsellor over the phone. So depending on the issue, they might then be assigned to a face to face counsellor. This programme is completely covered by the company. (EP20/M/30).

## Workplace Support for PMHC

Employers were additionally asked about organisational supports that they believe are needed at workplaces to facilitate hiring of PMHC. Suggestions by employers are described below.

## Workplace Mental Health Education and Peer Support Training

In relation to their inadequate knowledge and skills in supporting a PMHC, participants suggested educational workshops and trainings to equip them with literacy regarding mental illness, to be able to understand and respond better to PMHC and promote more inclusivity in the workplace.

Knowledge? Meaning uhm, because with knowledge we can understand, to understand means we probably learn how to deal with it. Yah because I always feel that without knowledge everyone is frightened, we do not know what’s, we’re always frightened of the unknown. (EP25/F/58).

They also suggested an element of de-stigmatisation by showcasing PMHC performing well at their jobs.

I think examples of real-life…examples of people maybe executive level, who have had mental health conditions, recovered, or have had to deal with working you know, like examples of that I think …not publicised but to serve as an example, I think it will really help. Or maybe through across all levels, I think that will help to break that. (EP05/F/35).

As some workplaces have a buddy system, they felt that a trained buddy would also be helpful to support the PMHC as they navigate through the organisation.

“How would a buddy help? Because they will show affections, they will show concern, they will always check on him, but definitely you need to attach a buddy which they know how to handle this type of people. (EP04/F/48)

### Availability of Professional Support

Others acknowledged that even with basic training, there may be certain situations that would best be handled by a professional. In such cases, an employer highlighted that having a mental health professional on call would be helpful.

If we could have a psychiatrist on standby 24 h and you know at any point if let’s say an episode kicks in and immediately, we call the psychiatrist and the psychiatrist can make a house call to the office you know or like an office call and come down and speak to the person and calm the person down, because they are medically trained on dealing with such situations right? (EP16/F/25).

Some employers shared that they have previously referred staff to professionals, who had presented with issues that they were unable to manage.

I do tie up with the community TOUCH then and then there’s SOS helpline. So I do call them and say that I do need help in this aspect. (EP22/F/44)

### Provision of Infrastructural Support

Employers suggested partnerships with employment support agencies who were better equipped to make the call on whether PMHC are ready for work and the suitability of a particular job for them.

Corporates will have to come and say, yes we are willing to employ them, yeah so at least we set up with our database. So that all these people can come and join them in an easier manner, otherwise they may not or, they go to interviews and they get rejected when they disclose all these things. (EP24/M/38).

So setting up an agency to manage this group of people who really can work or whatever would be much more important. (EP09/F/35)

Other employers recommended that in order to encourage employers to hire PMHC, tax rebates could be given.

So actually if your company employed some staff who has mental health conditions before, specified inside, so when you come to year-end tax, actually can rebate don’t know how many hundred thousand? That will be fantastic; I think the employer will be…trying? Somehow? (EP04/F/48).

## Discussion

The study showed that co-workers and employers generally believed that PMHC deserve to work, and employment is important to their recovery. However, a majority raised various concerns if PMHC were to be employed in their own workplace, save for several companies who had endorsed the fair employment practices agreement. Our findings reflect a variant of the ‘not in my backyard (NIMBY)’ phenomenon wherein co-workers and employers supported the ideology of the PMHC being included in the community in theory but not when they are in close proximity to them (Borell & Westermark, 2018). Similarly, previous studies have shown that positive attitudes towards diversity in the workplace were often not supported by their hiring practices (Burke et al., 2013; Jansson & Gunnarsson, 2018; Kaye et al., 2011).

The concerns about working with PMHC raised were in line with those elicited in previous research (Biggs et al., 2010; Dewa, 2014; Krupa et al., 2009; Shankar et al., 2014; Williams et al., 2016). To the best of our knowledge, this is the first qualitative study in Singapore exploring this topic and indicates the shared concerns in the Singapore context. These relate to fears that PMHC were unpredictable and dangerous, incompetent, and unable to get along with co-workers. Many employers and co-workers described their work as fast-paced and stressful, thus not suitable for PMHC. Unfortunately, several co-workers had negative experiences working with PMHC that supported their stereotypes (e.g., lashing out, absenteeism). Singaporeans often report being highly stressed and overworked, with 1 in 8 considering their stress levels unmanageable. Singapore has been ranked the “second most overworked city globally after Tokyo” (Gan, 2019). Thus, staff and employers felt that they did not have the capacity to hire PMHC whom they believed required more time and effort to train, supervise, provide psychosocial support, and present significant costs and risks to the company.

Another concern that appears persistently in stigma research in Singapore and emerges again in the workplace context in this study is the idea of “loss of face” (Tan et al., 2020a). “Face” represents one’s social identity and standing in the social hierarchy in Chinese and other Asian cultures and is thus important to business relations (Dong & Lee, 2007; Tan et al., 2020a). Co-workers and employers were concerned that PMHC’s poor workplace behaviour and quality of work would affect the company’s reputation, causing their employers to “lose face” to their customers and business partners. In addition, keeping up appearances of being busy, working late and long hours are part of the working culture in Singapore (Tang, 2018). Therefore, gaining entry into the workforce in Singapore or cities with similar working cultures maybe even more challenging for PMHC.

Due to the abovementioned concerns about working with PMHC and past negative encounters, many co-workers and employers felt that PMHC were only capable of carrying out limited, manual jobs such as cleaning, or backend tasks that were simplistic and repetitive, a stereotype which was described in earlier literature (Honey, 2004). The current study also noted that PMHC might have to “make up” for their mental illness by having exceptional qualities or accepting lower pay, further raising the barriers to employment. Employers and co-workers also pointed to companies whose social mission was to hire people with mental illness, indicating that non-competitive work activities are available and appropriate for people with mental illness. During the President’s Challenge in 2019, Singapore’s President Halimah Yacob addressed this issue by urging companies to offer a wider range of jobs, stating that PMHCs are well-qualified (Lee, 2019). Furthermore, job matching, i.e., matching a worker’s interests, values, and competencies, is associated with higher job satisfaction and longer tenure among PMHC (Mak et al., 2006; Resnick & Bond, 2001).

Many of the employers and co-workers indicated that they preferred PMHC to disclose their mental health condition for transparency, to be able to offer help if needed, be more understanding to PMHC, and be prepared for any incidents. However, they candidly admitted that disclosure could also affect PMHC’s chances of being hired, staying employed, or lead the PMHC to be subjected to prejudice and discrimination. The duality of findings regarding disclosure was also identified in Brouwer et al.’s study that included insights from human resource managers and mirrors the central dilemma facing PMHC as they enter the labour market (Brouwers et al., 2020). Although eliminating the declaration of mental health conditions during the job application process is likely to improve the chances of PMHC being hired, disclosure to employers and co-workers may benefit the PMHC. Apart from receiving understanding and support, the disclosure also helps the PMHC to be their authentic self; concealment made PMHC feel guilty, dishonest and exhausted (Brohan et al., 2012; Brouwers et al., 2020). Another important effect of disclosure is the increase in social contact with PMHC, which introduces powerful opportunities for stigma to be reduced (Dewa, 2014; Goldberg et al., 2005). It is possible that many of the employers and co-workers were already working alongside PHMC who were “passing”. Passing originates from Goffman’s (1963) work on stigma and refers to the processes of keeping a stigmatised identity concealed successfully. Passing allows the individual to successfully blend in, in the workplace and be treated the same as everybody else. (Brohan et al., 2012; Goldberg et al., 2005). However, as employers and co-workers were not aware of such contact, their biases were not dispelled.

With regard to existing workplace health supports, employers and co-workers described wellness workshops, informal or formal one-to-one sessions for PMHC to chat with their superiors, and employment assistance programmes by third-party providers. While these efforts represented an advancement in improving workplace health for PMHC, they indicated that more direct support was required if a PMHC was to be hired. Co-workers and employers admitted that their knowledge of mental illness was superficial, and they felt inept supporting a PMHC. Workshops imparting such knowledge and skills to co-workers and employers were thus desired. A systematic review of interventions targeting stigma in the workplace showed that educational interventions could be particularly effective in changing employees’ knowledge of mental disorders as well as helping behaviour (Hanisch et al., 2016). Two studies demonstrated spillover effects where desirable changes in behaviour occurred even though the intervention exclusively targeted knowledge or attitudes (Krameddine et al., 2013; Moffitt et al., 2014). Thus, interventions need not be complicated or costly but may have wide-ranging effects. Few studies in the literature have examined social contact with PMHC in workplace settings due to logistical challenges (Malachowski & Kirsh, 2013). However, co-workers and employers suggested that exposure to PMHC role models would facilitate the de-stigmatisation of mental health conditions. This is a promising strategy that, when combined with education, showed improvements in mental health literacy, attitudes towards people with mental illness, and attitudes towards mental health help-seeking among local university students (Shahwan et al., 2020; Subramaniam, Shahwan, et al., 2020; Tan, et al., 2020a, 2020b). Further research can explore the efficacy of such interventions in the workplace.

Employers and co-workers talked about having support from mental health experts to deal with crisis situations and help “manage” PMHC as they were not trained mental health professionals. Employment support providers are professionals who provide employment-related support, such as assessing for job suitability, vocational coaching, job placements (Vaingankar et al., 2020). Shankar et al. pointed that due to the diversity of the work context and individual differences among PMHC, tailored support will be appropriate, where employment support specialists work closely with managers to understand work demands and pressures and concerns about the PMHC’s condition and its likely impact on work (Shankar et al., 2014). The manager must then be involved in accommodations that may be needed and there must be periodic follow-up on how the PMHC is progressing in the workplace. The specialist should also be easily accessible by the manager for consultation. These supports to employers can encourage positive beliefs about the work capacity of PMHC and improve hiring and retention rates of PMHC in workplaces (Khalema & Shankar, 2014).

The above suggestions, dissemination of educational content, use of contact-based strategies, and provision of support for PMHC, employers, and employees are roles that are especially suited to community mental health providers. This is because they are well connected to members of the public, potential employers as well as social services in their community. They may also offer social opportunities to PMHC, which may influence recovery and improve chances of employment (Webber & Fendt-Newlin, 2017). A study of community reintegration programmes for adults with severe chronic brain injury and depression reported improvements in overall well-being, social outcomes, and employability (Geurtsen et al., 2008). PMHC’s participation in the community allows their increased contact with community members that play a role in de-stigmatisation. Community mental health providers may also ensure continuity of care for PMHC and provide support to employers when PMHC are discharged from institutional facilities. As community mental health care also encompasses viewing patients in their socio-economic context, these providers are trained and have the capacity to broker services and resources for PMHC (Thornicroft et al., 2016). Provision of these services may alleviate financial and housing difficulties that indirectly hinder employability (Cadorette & Agnew, 2017). Lastly, as suggested by some participants in this research, community mental health services may offer positions suitable to PMHC to work alongside mental health professionals. In a study comparing consumer-assisted and non-consumer-assisted case management with standard clinic-based care, all three programs yielded the same general pattern of improvement among clients over time for symptoms, health care satisfaction, quality of life and social network behavior (Rivera et al., 2007). Incentivising employers by providing tax credits and salary support for hiring PMHC were also suggested by employers and co-workers. Many countries offer tax credits and other financial incentives to employ workers with disabilities, which emerging research indicates enhance employment of PMHC (Heaton, 2018). Other strategies identified in the literature were the provision of financial assistance to cover the cost of accommodations that would otherwise impose “undue hardship” on business operations (Hollenbeck, 2015; Khalema & Shankar, 2014). It should be noted, however that direct costs associated with reasonable accommodation for PMHC are often nominal. The most common reasonable accommodations include flexible scheduling, enhanced training and supervision, and modified job duties (Delman et al., 2017). Overall, evidence shows that such accommodations enhance productivity when supervisors are knowledgeable about assessing and providing accommodation requests (Schultz et al., 2011). There are also local data to support business sustainability of investing in workplace accommodations. For example, a 2017 survey of 505 companies by NCSS found that for every S$1 invested in workplace adjustment (like flexible work arrangements, job redesign, peer training) to support PMHC, it generated an average return of S$5.60 through a reduction in absenteeism and medical claims as well as an increase in productivity (Ong, 2020).

Overall, the research uncovered a barrage of unmet needs and gaps to fill in the employment of PMHC. However, policy changes have taken place recently and nationwide initiatives and political figures have improved the visibility of mental health conditions. The nationwide campaign Beyond the Label was launched in September 2018 by NCSS to destigmatise mental health conditions. NCSS also published a toolkit to support hiring and retaining PMHC in workplaces (NCSS, 2019). More recently, an advisory on mental well-being at workplaces was jointly issued by the Ministry of Manpower, Singapore National Employers Federation, and the National Trades Union Congress (MOM, 2020). In addition, a Workwell Leaders Workgroup that champions workplace mental well-being brought together some 50 CEOs across private, people, and public sectors in Singapore to participate in a dialogue session on 4^th^ December 2020. This session aimed to prioritise mental health and share ideas on best practices for mental health initiatives in the workplace (WorkWellLeaders, n.d.), indicating a start to a culture shift that heralds an optimistic future for PMHCs in the labour force.

### Limitations and Strengths of the Study

The participants were obtained through referrals from the study team and other colleagues. These individuals who responded and voluntarily participated may have more favourable attitudes towards the employment of PMHC that may have motivated their participation. Only participants who were able to speak English were included in this study. Participants who are not English speakers may have other views that were not captured in this study. Social desirability bias may have affected some of the responses and led to underreporting of overtly prejudiced or biased attitudes. Next, responses by employers and co-workers with and without actual prior contact with PMHC were mixed. Contact with PMHC was also based on self-report which could have been based on either conjecture or actual knowledge that the PMHC was diagnosed and/or seeking help. Thus, among those without actual social contact, responses were based on hypothetical scenarios. On the other hand, those that were based on actual experience may have been influenced by serious workplace incidents. Therefore, neutral and positive experiences of working with PMHC whose conditions were not known to them, whom Goffman referred to as “passing” were not captured. The data of this study were also obtained before the declaration of mental illness in job application was eliminated and other major milestones in mental health promotion in Singapore were launched (e.g., Beyond the Label campaign, President’s Challenge and WorkWell Leaders’ dialogue). These initiatives may have resulted in improved attitudes towards PMHC. Further research is needed to update local knowledge of attitudes towards PMHC, and how new policies have impacted organisations. Despite the limitations, this study represents early efforts in Singapore to understand the needs of employers and co-workers from various industries in supporting a PMHC. The data also represents universal challenges in provision of jobs for PMHC in an urban Asian setting and how guidelines from other countries may be applied with local adaptations.

## Data Availability

Participants of this study did not agree for their data to be shared publicly. Supporting data is therefore not available.
